# Dynamic Expression Profile, Regulatory Mechanism and Correlation with Egg-laying Performance of *ACSF* Gene Family in Chicken (*Gallus gallus*)

**DOI:** 10.1038/s41598-018-26903-6

**Published:** 2018-05-31

**Authors:** Weihua Tian, Hang Zheng, Liyu Yang, Hong Li, Yadong Tian, Yanbin Wang, Shijie Lyu, Gudrun A. Brockmann, Xiangtao Kang, Xiaojun Liu

**Affiliations:** 1 College of Animal Science and Veterinary Medicine, Henan Agricultural University, Zhengzhou, 450002 China; 2 Henan Innovative Engineering Research Center of Poultry Germplasm Resource, Zhengzhou, 450002 China; 3 International Joint Research Laboratory for Poultry Breeding of Henan, Zhengzhou, 450002 China; 4 Albrecht Daniel Thaer-Institute of Agricultural and Horticultural Sciences, Humboldt-Universit€at zu Berlin, Invalidenstraße 42, Berlin, 10115 Germany

## Abstract

Acyl-CoA synthetases (ACSs) are responsible for acyl-CoA synthesis from nonpolar hydrophilic fatty acids and play a vital role in many metabolic processes. As a category of ACS isozymes, members of ACS family (ACSF1-3) participate in lipid metabolism; however, their expression patterns, regulatory mechanisms and effects on egg-laying performance in chicken are poorly understood. Our *in vivo* and *in vitro* studies showed that *ACSF1-3* genes were extensively expressed, and their expression levels changed dynamically in the liver among different development stages. Moreover, *ACSF1* expression was upregulated and *ACSF2* expression was downregulated by estrogen, but *ACSF3* showed no response to estrogen treatment. The regulatory effect of estrogen on *ACSF1* expression was mediated via ERα. The *ACSF2* was highly expressed in the liver in peak-laying hens compared with pre-laying and late-laying hens, and also highly expressed in the liver continued egg-laying hens compared with inactive egg-laying hens. It is suggested that hepatic *ACSF2* expression level might relate to egg-laying performance in chicken. In conclusion, the expression of *ACSF1* was upregulated by estrogen via ERα, and the expression of *ACSF2* was downregulated by estrogen and might be related to egg-laying performance in chicken.

## Introduction

Fatty acids (FA) play crucial roles in organisms, for example, offering a source of energy, activating the synthesis of bio-membranes as well as participating in metabolic pathways^1,2^. As members of the lipid group of molecules, FAs have chain lengths ranging from two carbons, for acetate, to more than 30 carbons for some waxes and plant lipids. However, they are chemically fairly inert and need to undergo activation into acyl-CoA supplemented with Mg^2+^, ATP and CoA in liver cells, for the formation of complex lipids such as triglycerides, phospholipids and cholesterol esters. Acyl-CoA synthetases (ACSs) are involved in this process. ACSs are located in the endoplasmic reticulum and mitochondrial outer membrane, and are a major category of enzymes that catalyse nonpolar hydrophilic FA into acyl-CoA^3,4^. Depending on the sequence identity and substrate preference regarding the chain lengths of fatty acids, human ACSs are grouped into 26 ACS isozymes, which are divided into six families, namely, the ACS short-chain family (ACS), ACS medium-chain family (ACSM), ACS long-chain family (ACSL), ACS very long-chain family (ACSVL), ACS bubblegum family (ACSBG) and ACSF family (ACSF)^5,6^. As one of the ACS isozymes, ACSF has been proven to be involved in FA and cholesterol synthesis^7^, metabolism-related disease^8^, as well as egg laying rate in poultry^6^.

In great contrast to the case in mammals, little or no fatty acid synthesis occurs in adipose tissue in chicken; instead, liver is extremely important in lipid synthesis, degradation and transport^9,10^. It is widely known that estrogens are crucial for development and reproductive performance. It has also been proved that lipid metabolism in chicken liver is strongly affected by estrogen^11^, which exerts significant effects in regulating lipogenic genes equipped with either classical estrogen response elements (ERE)^12^ or nonclassical AP1 site^13^ via direct or indirect binding to estrogen receptors (ER) including ER alpha (ERα), ER beta (ERβ) and a G-protein-coupled receptor (GPR30)^14–16^. For the classical ERE pathway, the ligand binding domain of ERα and ERβ binds as homodimers or heterodimers to ERE in the promoter of target genes and recruits a variety of transcriptional cofactors to produce transcription initiation complex, leading to the activation of the enhancer residing in the regulation regions and the promotion of targets transcription. As a member of the G protein-coupled receptor superfamily, GPR30 mediates estrogen-dependent kinase activation and transcriptional responses. GPR30 could produce biological effects through binding to the estrogen or its ramification for the purpose of regulating targets transcription in a rapid nongenomic signaling. It could activate the multiple cellular kinase pathways, such as PI3K, Elk-1, SRF, MAPK and so on, in order to indirectly regulate a series of genes transcription and mediate many biological functions of estrogen^16^. About the nonclassical AP1 pathway, ER has been proved to affect gene expression from promoters containing an AP1 site with the action of transcription factors Fos and Jun^15,17,18^. Interestingly, there are substantial differences in transcription in those genes containing an AP1 site in their promoters; some are negatively regulated by estrogen while bound to ERα, like human prolactin^19^, but others are positively regulated by it while bound to ERβ, such as progesterone receptor gene^20^. The estradiol antagonists tamoxifen and ICI 182,780 to some extent stimulate transcription via the AP1 reporter in the presence of ERβ^13^.

Though ACSFs have been extensively studied in mammals, little is known about their effects and specific regulatory mechanism in avian species. Interestingly, two recent reports descripted that the ACSF2 was responsible for the laying performance in geese, with ACSF2 expression being decreased in the ovary of high-producing geese. It was asserted that its lower expression may promote laying performance by inhibiting granulosa cell apoptosis and facilitating follicular development in geese^6^.

The Lushi green-shelled-egg chicken is a native chicken breed in China. It lays its first egg at an average age of 21 weeks and reaches its peak rate of egg production at about 28 weeks old. The average annual number of eggs is 150–180 with an average weight of 47 g.

In the present study, the Lushi green-shelled-egg chicken was used to study on ACSFs with the following objectives: 1) to evaluate the expression profiles of *ACSFs* in various tissues and developmental stages, 2) to investigate the regulatory mechanism of *ACSF* expression and 3) to determine the correlation of *ACSF* expression with egg-laying performance in chicken.

## Results

### Conserved synteny analysis for the genomic region of the *ACSF1/AACS* gene

**Figure 1:**
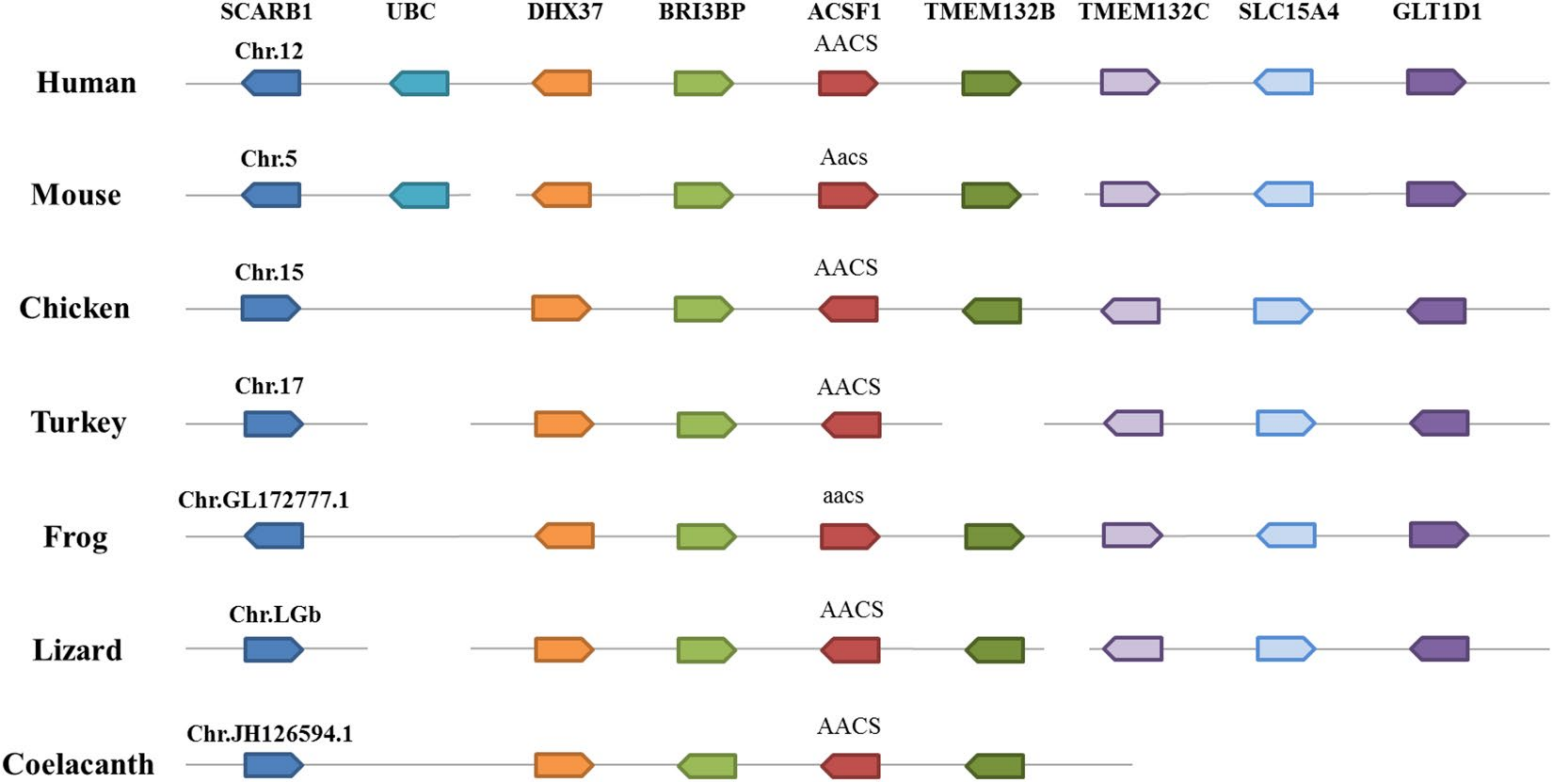
Conserved synteny for the genomic region of *ACSF1*/*AACS* gene. The species are listed on the left of the chromosomes, and the gene symbols are listed on the top. The genes presented by different color boxes in the same column mean existing in the according species. The direction in which the boxes of the different genes are pointing represents their direction of transcription. The detailed genomic locations of genes are listed in Supplementary Table S2.

In human, *ACSF1* is also known as acetoacetyl-CoA synthetase (*AACS*). However, only *AACS* has been identified in chicken and other species by sequence mining of NCBI and other public genome databases. To evaluate whether the existed chicken *AACS* also means *ACSF1* in chicken, a syntenic analysis of the *AACS* neighbouring genes was performed in seven representative genomes including human, mouse, chicken, turkey, frog, lizard and coelacanth. The results showed that *AACS* in chicken and other species is positioned at the same locus of a conserved genomic region arranged with common genes including *UBC*, *DHX37*, *BRI3BP*, *TMEM132B*, *TMEM132C*, *SLC15A4* and *GLTD1* among species. The detailed genomic locations of these genes are given in Table S2. This demonstrated that, as in human, the *AACS* gene is also the *ACSF1* gene in chicken and other species (Fig. 1).

### Amino acid sequence alignment and phylogenetic analysis of ACSFs

Multiple alignment analysis of amino acid sequences of ACSF1, ACSF2 and ACSF3 among eight species from mammals, reptiles, avians, amphibians and fish was performed by DNAMAN. The results revealed that ACSF1, ACSF2 and ACSF3 shared 82.05%, 70.10% and 56.67% identity among species (see Supplementary Fig. S1a–c). The amino acid sequences among ACSF1, ACSF2 and ACSF3 in chicken shared low identify from 12.97% to 15.45% (see Supplementary Fig. S1d).

**Figure 2:**
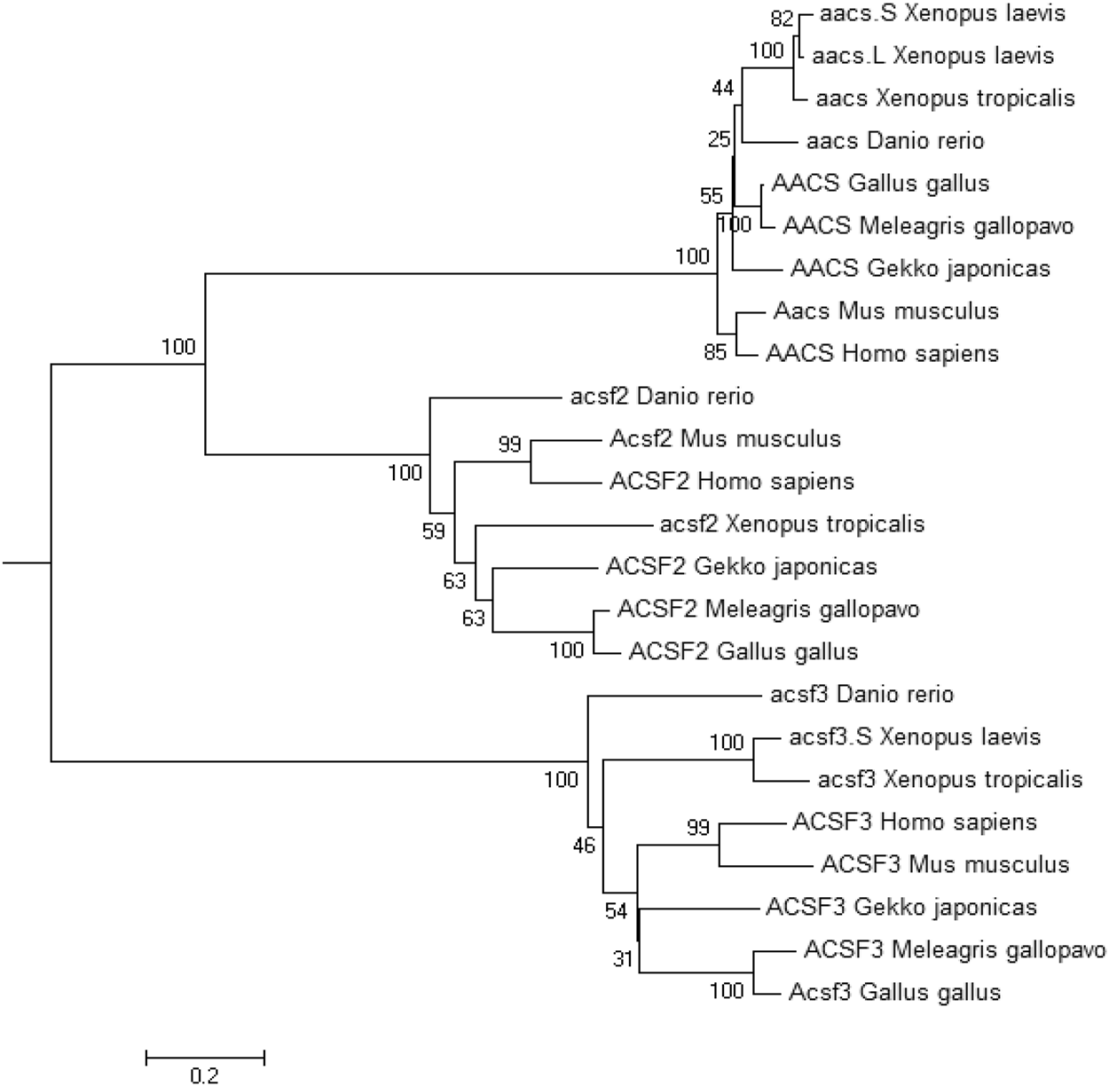
Phylogenetic analysis of ACSF1, ACSF2 and ACSF3 amino acid sequences. The amino acid sequences name and NCBI accession numbers used in the phylogenetic tree construction were as follows: hs AACS, NP_076417.2; mm Aacs, NP_084486.1; gj AACS, XP_015276338.1; gg AACS, NP_001006184.1; mg AACS, XP_010718535.1; xl aacs.S, XP_018099672.1; xl aacs.L, XP_018117990.1; xt aacs, NP_001039244.1; dr aacs, NP_957303.1; hs ACSF2, NP_001275897.1; mm Acsf2, NP_722502.1; gj ACSF2, XP_015266223.1; gg ACSF2, XP_015151000.1; mg ACSF2, XP_010719920.1; xt acsf2, XP_002939621.2; dr acsf2, NP_001132910.1; hs ACSF3, XP_011521244.1; mm Acsf3, NP_659181.2; gj ACSF3, XP_015262215.1; gg ACSF3, XP_425134.4; mg ACSF3, XP_010716483.2; xl acsf3, NP_001086314.2; xt acsf3, NP_001121382.2; dr acsf3, XP_021329817.1.

Amino acid sequences of ACSF subtypes from different species including mammals (human, mouse), avians (chicken, turkey), reptile (gecko), amphibians (African clawed frog, Western clawed frog) and fish (zebrafish) were retrieved from GenBank. Based on the alignment of the amino acid sequences, a phylogenetic tree was constructed (Fig. 2). The results indicated that the ACSF subtypes were conserved among species and clustered into two big clades then ran to a rooted clade, so that ACSFs might be evolutionally derived from a uniform ancient gene, and ACSF1 and ACSF2 were clustered into the same big clade, manifesting that ACSF1 and ACSF2 were likely orthologous.

Protein functional domain analysis showed that the AMP-binding domain, which is a characteristic feature of acetyl-CoA synthases, was evolutionarily conserved in all chicken ACSF subtypes (see Supplementary Fig. S2), suggesting their importance for the lipid metabolism.

### Tissue expression profiles of *ACSFs* in chicken

**Figure 3:**
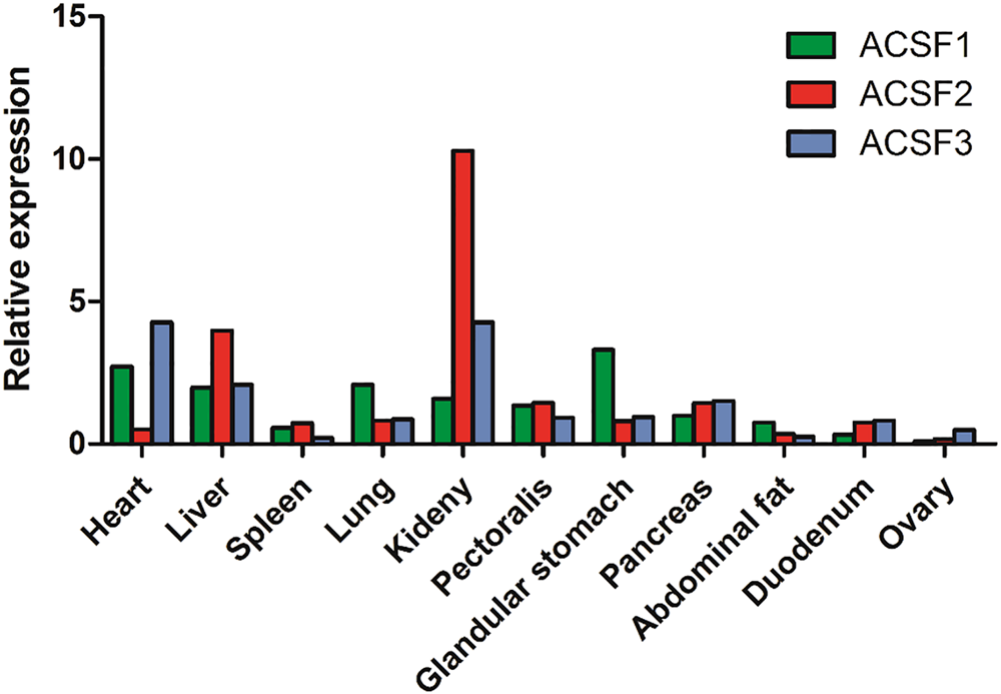
Tissues distribution of *ACSF1*, *ACSF*2 and *ACSF3* in laying hens. The housekeeping gene *β-actin* was used as an internal standard to estimate mRNA relative expression. The 11 cDNA pools for different tissues are composed of the equal amount of 8 samples.

The specific expression patterns of *ACSF* subtypes in various tissues including heart, liver, spleen, lung, kidney, pectoralis, glandular stomach, pancreas, abdominal fat, duodenum and ovary of 30-week-old chickens were determined by qPCR analysis. The results indicated that *ACSF* subtypes were broadly expressed in all tested tissues, and especially highly expressed in kidney and liver (Fig. 3).

### Expression patterns of *ACSFs* in the liver at different developmental stages of chicken

**Figure 4:**
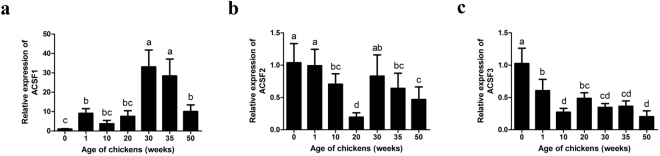
The relative mRNA expression level of chicken *ACSF1* (**a**), *ACSF*2 (**b**) and *ACSF3* (**c**) in liver tissues at different stages. The housekeeping gene *β-actin* was used as an internal standard to estimate mRNA relative expression. All data were shown as fold change compared with gene expression in 0 weeks group. Data were represented as Mean ± SD (n = 8). Values with different lowercase mean significant difference (p < 0.05).

Liver is the key organ involved in lipid metabolism in chicken. To further understand the function of ACSFs in lipid metabolism in chicken, the expression patterns of *ACSF* subtypes in liver of chicken at different developmental stages, including 1 day old, and 1, 10, 20, 30, 35 and 50 weeks old, were analysed using qPCR. The results revealed that the *ACSF1* mRNA expression level was lower in the early stage of growth and development, and then increased significantly after sexual maturity, but decreased again in the late laying period (Fig. 4a). The mRNA expression levels of *ACSF2* and *ACSF3* peaked just after hatching and then gradually fell down along with chicken growth and development (Fig. 4b,c). For *ACSF3*, its expression decreased to the lowest level at 10 weeks old and then remained at similar levels during the pre-laying, peak laying and late laying stages (Fig. 4c). For *ACSF2*, however, its expression dropped to the lowest level at 20 weeks old, and then significantly increased as sexual maturation arrived, after which it gradually decreased until the late laying period (Fig. 4b).

### Effects of 17β-estradiol on the mRNA expression of chicken *ACSFs in vivo* and *in vitro*

**Figure 5:**
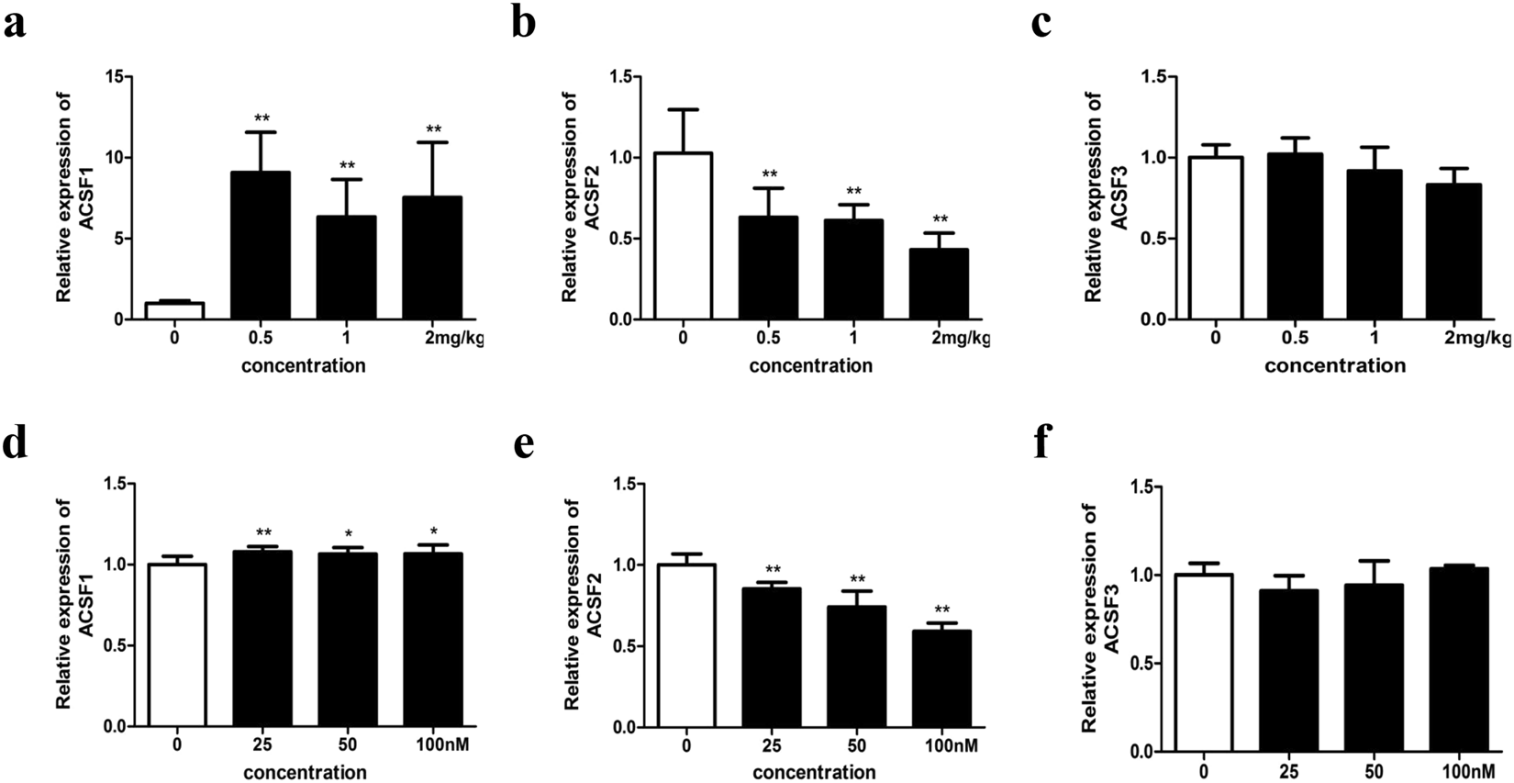
Effects of different doses of 17 β-estradiol on the mRNA expression of *ACSF* subtypes in chicken liver tissues and primary hepatocytes. (**a**–**c**) Effects of different doses of 17 β-estradiol on the mRNA expression of *ACSFs* in chicken liver tissues. (**d**–**f**) Effects of different doses of 17 β-estradiol on the mRNA expression of *ACSFs* in chicken primary hepatocytes. The housekeeping gene *β-actin* was used as an internal standard to estimate mRNA relative expression. All data were shown as fold change compared with gene expression in 0 nM or 0 mg/kg group. For each treatment in chicken liver tissues, data were represented as Mean ± SD (n = 10); for each treatment in chicken primary hepatocytes, data were represented as Mean ± SD (n = 6). *Significant differences (P < 0.05), **highly significant differences (P < 0.01).

Estrogen is generally considered to be a major factor regulating the expression of genes related to lipid metabolism in chicken liver. To understand the mechanism regulating *ACSF* expression, the mRNA expression levels of the three *ACSF* subtypes were investigated in the livers of chickens challenged with different doses of 17β-estradiol. The findings showed that the mRNA expression of the *ACSF* subtypes exhibited different responses to the 17β-estradiol treatment. The mRNA expression level of *ACSF1* exhibited a significant increase in a dose-dependent manner after 17β-estradiol treatment for 12 h (Fig. 5a); in contrast, that of *ACSF2* was significantly decreased (Fig. 5b). No change occurred in *ACSF3* (Fig. 5c). To further validate the different regulatory effects of estrogen on the expression of the three *ACSF* subtypes *in vitro*, chicken primary hepatocytes were treated with different concentrations of 17β-estradiol for 12 h. The results showed that the mRNA expression level of *ACSF1* was significantly increased (Fig. 5d), that of *ACSF2* was significantly decreased in a dose-dependent manner and that of *ACSF3* showed no change (Fig. 5e,f), which almost exactly matched the findings for the *ACSF* gene family in chicken liver tissues. Meanwhile, as a marker gene of estrogen response, the mRNA expression of *ApoVLDL II* was monitored. The *ApoVLDL II* mRNA expression was significantly increased in a dose-dependent manner in both *in vitro* and *in vivo* studies, indicating that estrogen possessed biological activity and functioned physiologically in chicken livers (see Supplementary Fig. S3a) and embryonic primary hepatocytes (see Supplementary Fig. S3b).

### Effects of the co-treatment of 17β-estradiol and ER antagonists MPP, tamoxifen, and ICI 182, 780 on *ACSFs in vitro*

**Figure 6:**
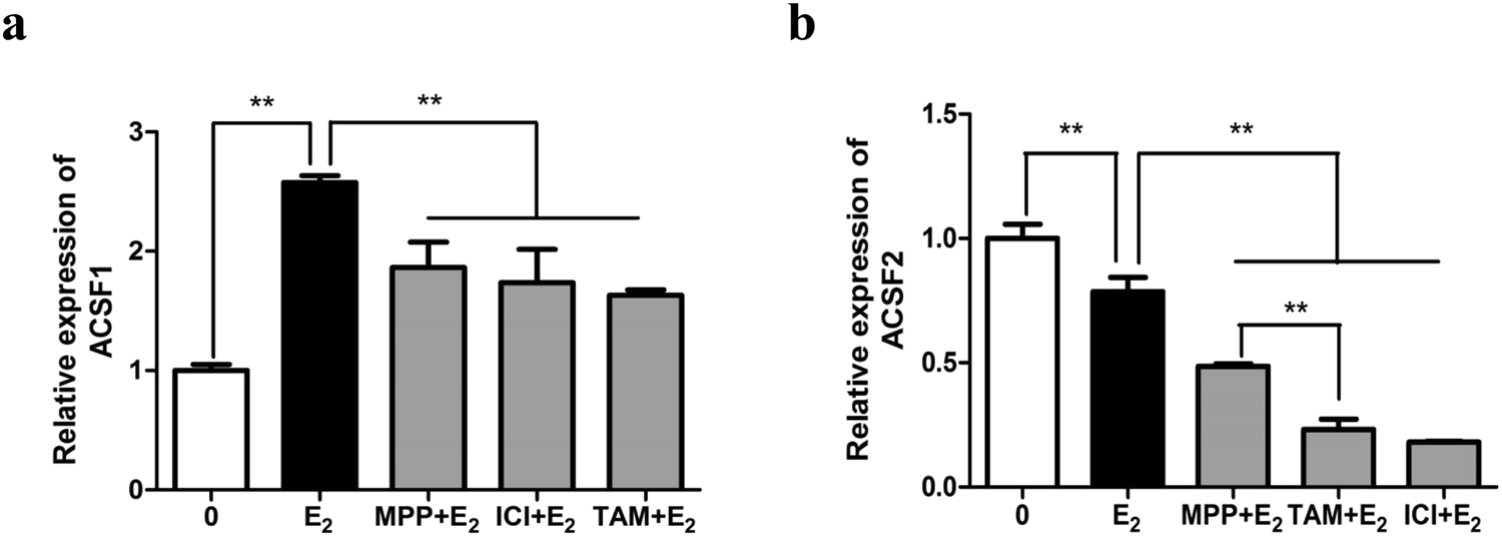
Effects of co-treatment of 17β-estradiol and ER antagonists MPP, tamoxifen, and ICI 182, 780 on the mRNA expression of *ACSF1* (**a**) and *ACSF2* (**b**) in chicken primary hepatocyte, respectively. E_2_:17β-estradiol (100 nM); ICI: ICI 182,780 (1 μM), TAM: tamoxifen (1 μM), and MPP:(1,3-bis(4-hydroxyphenyl)-4-methyl-5-[4-(2-piperidinylethoxy)phenol]-1H-pyrazoledihydrochloride) (1 μM). The housekeeping gene *β-actin* was used as an internal standard to estimate mRNA relative expression. All data were shown as fold change compared with gene expression in 0 nM group. For each treatment, data were represented as Mean ± SD (n = 6). *Significant differences (P < 0.05), **highly significant differences (P < 0.01).

To further define the specific ER that mediates the effect of estrogen on *ACSF1* and *ACSF2* expression, chicken primary hepatocytes were treated with 17β-estradiol together with various ER antagonists. The results indicated that, as expected, *ACSF1* mRNA expression presented a highly significant increase when the primary hepatocytes were treated with 100 nM 17β-estradiol. However, highly significant decreases occurred while combined treatment of 17β-estradiol and an ER antagonist, MPP, TAM or ICI, was carried out (Fig. 6a). As for *ACSF2*, its expression was significantly decreased when the cells were treated with 100 nM 17β-estradiol in comparison with the case for the untreated control. The addition of 1 µM MPP did not disturb the effect of estrogen on *ACSF2* expression. However, supplementation of 1 µM TAM or ICI in the cell culture medium led to a further reduction of *ACSF2* expression (Fig. 6b). This implied that the expression of both *ACSF1* and *ACSF2* are regulated by estrogen, but through different mechanisms.

### Correlation of *ACSFs* expression with laying performance

**Figure 7:**
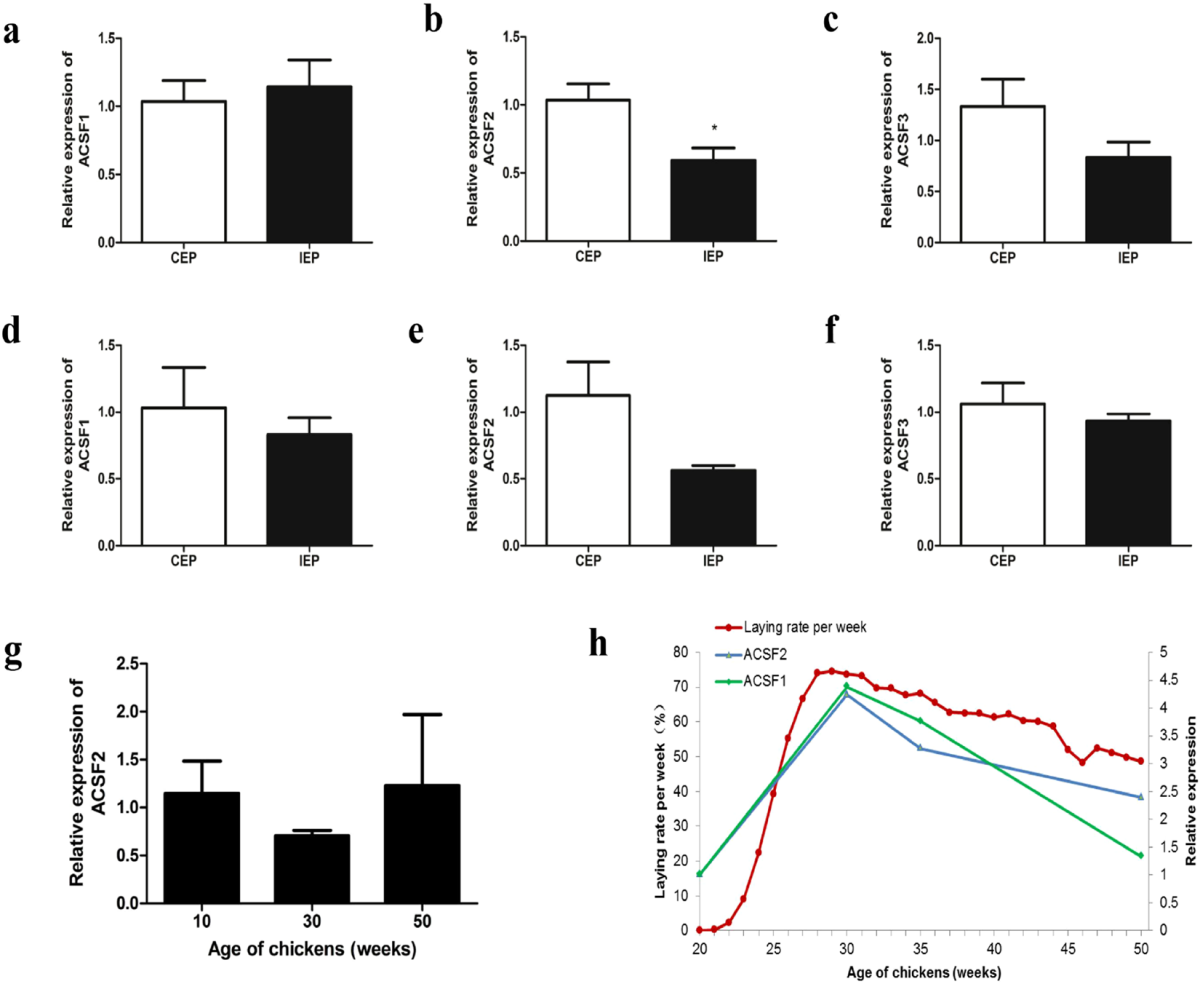
Correlation between chicken *ACSF2* mRNA expression and laying performance. (**a**–**c**) Expression pattern of *ACSFs* in livers from CEP and IEP. (**d**–**f**) Expression pattern of *ACSFs* in ovaries from CEP and IEP. (**g**) Expression pattern of *ACSF2* in ovaries from different developmental stages. (**h**) The changing trends of *ACSF2* expression and laying rate per week in Lushi green-shelled-egg chickens. The housekeeping gene *β-actin* was used as an internal standard to estimate mRNA relative expression. All data were shown as fold change compared with gene expression in CEP. Data were represented as Mean ± SD (n = 8). *Significant differences (P < 0.05), **highly significant differences (P < 0.01).

By analysing the different expression of *ACSFs* between CEPs and IEPs, we found that the expression levels of *ACSF1* and *ACSF3* were not significantly different between the two groups in their livers, but *ACSF2* expression was remarkably higher in the livers of CEP (Fig. 7a–c). Moreover, ovarian *ACSFs* had no response in both groups (Fig. 7d–f), which suggested that it is hepatic ACSF2 that is involved in chicken egg production. Similarly, *ACSF2* was stably expressed in ovaries in three major developmental stages, namely, the early-, peak- and late-laying periods (Fig. 7g). Previous results concerning the differential expression of *ACSF2* in chicken livers at different developmental stages identified its high expression during the peak laying period, but clearly decreased expression in either the early or the late laying period, improving our confidence in the notion that hepatic *ACSF2* expression was associated with laying performance. From the egg production records, Lushi green-shelled-egg chickens had an average age of first-egg laying at 21 weeks and a peak laying rate at 28 weeks. We further comparatively analysed the correlation between the dynamic expression pattern of *ACSF2* mRNA and the egg production curve ranging from 20 to 50 weeks of age in Lushi green-shelled-egg chickens, which indicated that egg production varied in association with *ACSF2* mRNA expression (Fig. 7h). Therefore, it can be asserted that hepatic *ACSF2* affects egg production in chicken.

## Discussion

ACSF family plays a crucial role in lipid metabolism in mammals^5^. However, to the best of our knowledge, no information on chicken ACSF family has been reported until now. In the present study, we first demonstrated the existence of *ACSF1* in the chicken genome, according to consensus conserved genomic synteny of *AACS* between chicken and other species. Multiple nucleotide sequence comparison among different species indicated that *ACSF1* and *ACSF2* shared relatively high identity, whereas *ACSF3* showed relatively low identity. Amino acid analysis revealed that three highly conserved motifs in chicken ACSF1—motif I: YSSGTTGAPK, motif II: HGDYCKINPKTGGVVMLGRSDGTLNPNGVRFGSSEIY and motif III: PYTLNGKKVE; five in chicken ACSF2, motif I: FTSGTTGSPK, motif II: TGDIATLDEHGYCRIIGRCKDMIIRGGENIYPAEIE, motif III: YGTTE, motif IV: APLYH and motif V: PLTVSGKIQK; and three in ACSF3, motif I: YTSGTTGRPK, motif II: YGMTE and motif III: LPLHH—were identified by comparison with human ACSF1, ACSF2 and ACSF3, respectively. The conserved motifs probably have important functions in substrate binding and/or catalysis^5^. Protein functional domain analysis of chicken ACSFs identified a conserved AMP binding site, consistent with the notion that ACSs are finely regulated at the transcriptional level via cyclic AMP (cAMP)-driven trans-activation^21^. Numerous prokaryotic and eukaryotic enzymes, which appear to act via ATP-dependent covalent binding of AMP to their substrate, share a region of sequence similarity, such as long-chain fatty acid Co-A ligase and ACSs^22^. Notably, these conserved motifs of ACSFs are located in their corresponding AMP binding regions. These results suggest that ACSF1 and ACSF2 could have similar evolutionary history and function while ACSF3 is more divergent, which may have gained another novel function due to whole-genome duplications^23^. Moreover, the conserved residues or domains may be important for substrate binding^24^.

The temporal and spatial expression pattern analysis of *ACSF1*, *ACSF2* and *ACSF3* indicated that they are highly expressed in the liver and kidney of laying hens, which is consistent with previous findings on the *ACSF2* mRNA expression level in goose^6^ and ACSF3 protein level in mouse^25^. Moreover, in chicken, the kidney and liver are the organs in which lipids are efficiently metabolised, especially the liver. The expression analysis of *ACSF* genes in livers from different stages indicated that *ACSF1* and *ACSF2* presented higher expression in chicken liver in the peak laying period (30 weeks old) than in the early (20 weeks old) and late laying periods (50 weeks old), whereas no changes were seen in *ACSF3*. Lipid synthesis and metabolism drastically increased from the early to the peak laying period, in order to synthesize the nutrients needed during embryonic development for the formation of the egg yolk. Therefore, we speculated that *ACSFs* probably participate in avian lipid metabolism, and that *ACSF1* and *ACSF2* play dominant roles in this, while *ACSF3* does not. Further studies focusing on the function of the chicken *ACSF* gene family in the lipid metabolism of the kidney are needed.

Estrogen plays a vital role in sexual maturity and the development of female reproductive activity, and it can regulate the lipid metabolism of hens during the laying period. Liver, as an organ that is of extreme importance in lipid metabolism and targeted by estrogen, was used to study the effects of estrogen on ACSFs, as far as their high expression in the liver of peak laying hens was concerned. We optimized the doses of estrogen used for both *in vivo* and *in vitro* studies according to the previous reports^26–29^, our preliminary experiments and related work^30^. Our results revealed that *ACSF1* and *ACSF2* were positively and negatively regulated in a dose-dependent manner by estrogen, respectively, but no alteration was found in *ACSF3*. At the same time, upon the treatment of chicken primary hepatocytes with different concentrations of estrogen, a gradual increase of *ACSF1* mRNA and a gradual decrease of *ACSF2* mRNA were found, but there was no change in* ACSF3* mRNA. As hens mature sexually, plasma estrogen concentrations gradually rise, with remarkable increases when hens are in the age range from 16 to 20 weeks old, and remain high for the next several weeks for egg production, and then decline until stop laying^31^. The higher expression of *ACSF1* in estrogen-treated livers and cells was consistent with that in the peak laying period compared with that in either the early or the late laying period, while the expression of *ACSF3* was also unchanged, and *ACSF2* showed the lower expression in estrogen-treated livers and cells but higher expression in the peak laying period. With the arrival of the peak laying period, some hormones apart from estrogen, such as follicle-stimulating hormone (FSH) and luteinizing hormone (LH), were physiologically increased in line with physiological processes by a large margin. These other hormones rather than estrogen might explain the higher expression of *ACSF2* at 30 weeks old but its lower expression after estrogen treatment.

It was reported that estrogen has a wide range of functions in regulating lipogenic genes with either ERE or AP1 site by directly or indirectly binding to ER, including nuclear receptors (ERα, ERβ) and membrane receptor (GPR30). To investigate the molecular regulatory mechanism of estrogen on *ACSF1* and *ACSF2*, further analysis of their promoters was performed, which showed that a few nonclassical ERE sites were present in *ACSF1*, and an exclusive AP1 site was present in *ACSF2*. Selective estrogen receptor antagonists, such as MPP, TAM and ICI, are another approach for studying the functions of receptors besides gene knockout^32–34^. When bound to an antagonist, the ER does not interact with coactivators and in turn does not activate transcription^13^. Since MPP is highly selective for ERα, not only are TAM and ICI the primary antagonists for ERα and ERβ, but they also act as agonists on GPR30 owing to high affinity for it. *ACSF1* mRNA expression was significantly increased in the mere estrogen-treated primary hepatocytes, but was highly significantly decreased upon applying combined treatment of estrogen and MPP, TAM or ICI. Therefore, chicken *ACSF1* expression was shown to be regulated by estrogen mediated via ERα. *ACSF2*, with an AP1 site in its promoter, was correspondingly decreased after treatment with estrogen alone, but interestingly was enormously decreased again after MPP, TAM and ICI treatment. There was evidence that substantial transcriptional differences exist in these genes containing an AP1 site in their promoters, of which some are negatively regulated by estrogen while binding to ERα, but others are positively regulated by it while binding to ERβ^35^. At present, to our knowledge, there is no evidence to support the dynamic regulation of the genes with an AP1 site while binding to GPR30. Estrogen antagonists, namely, tamoxifen and ICI 182,780, can stimulate transcription via the AP1 reporter in the presence of ERβ^13,36,37^. When challenged with estrogen, *ACSF2* mRNA was observably decreased, suggesting that estrogen could regulate* ACSF2* via binding to ERβ. However, upon treatment with respective MPP, TAM and ICI in combination with estrogen, a remarkable decrease occurred in *ACSF2* again, which conflicted with the above-mentioned notion that TAM and ICI can stimulate transcription via the AP1 reporter in the presence of ERβ. The limitation of gene expression regulation from a number of factors and diversification of estrogen regulation patterns at the transcription level provide many possible explanations for specific molecular machinery of estrogen on *ACSF2* expression, for example, induction by estrogen of transcription factors and methylation, mediated by GPR30. This shows that the molecular machinery that allows estrogen to control *ACSF2* expression is not completely understood and warrants further study.

ACSs are responsible for acyl-CoA synthesis from nonpolar hydrophilic fatty acids and play an important role in many metabolic processes, including the synthesis of cholesterol and other lipid molecules as well as the tricarboxylic acid cycle. FA activation comprises a two-step and ATP-dependent reaction catalysed by ACS enzymes with the formation of an intermediate acyl-AMP, which is then converted to acyl-CoA^4^. The length of the carbon chain of the fatty acids defines the substrate specificity for different ACSs^4,38^. It has been suggested that ACSF1 catalyses a single condensation of acyl-CoA and malonyl-CoA to acetoacetyl-CoA^39^. As was previously reported, ACSF2 robustly activated the eight-carbon medium-chain fatty acid octanoate in *ACSF2*-overexpressing cells^5^. Besides, lower expression of *ACSF2* was proved to reduce the formation of acetyl-CoA, which was used to participate in the tricarboxylic acid cycle to promote the release of ATP; this consequently contributed to the inhibition of ATP release, and then the decrease of granulosa cell apoptosis, ultimately increasing the laying performance in goose^6^. Interestingly, our results identified that there were no changes in *ACSF2* mRNA expression in ovaries, but significant changes in livers from chickens with continued egg production, inactive egg production, and different physiological chickens. Therefore, we suggested that not ovarian but hepatic *ACSF2* is greatly involved in chicken egg production. The difference that *ACSF2* showed a lower expression in the ovary high egg production geese but no change in the ovary of both continued and inactive egg production hens, instead, a higher expression in the liver of the former hens might imply diverse roles of *ACSF2* in laying performance between chicken and goose, which requires further study. In terms of ACSF3, it was previously tentatively identified as an acyl-CoA synthetase, which prefers a substrate for lignoceric acid, a 24-carbon very long-chain fatty acid. A novel function of ACSF3 has been demonstrated, in that it also serves as a mitochondrial malonyl-CoA synthetase to generate intramitochondrial malonyl-CoA from malonate in mammals^25,40^, which can then be used by fatty acid synthase to generate long-chain fatty acids, or be used for chain elongation of fatty acids^41^. We concluded that estrogen, binding to ERα through ERE, positively regulated *ACSF1* expression, which serves as a catalyst for acetoacetyl-CoA from a single condensation of acyl-CoA and malonyl-CoA, thereby giving rise to lipogenesis; estrogen, via the AP1 pathway, negatively regulated *ACSF2* expression, which has access to the activation of eight-carbon medium-chain fatty acids, affecting lipid metabolism and reproductive performance.

## Conclusion

In conclusion, the expression of *ACSF1* is significantly increased in laying hens in comparison with that in pre-laying juveniles, and positively regulated by estrogen via binding to ERα. Owing to the expression of *ACSF2* being negatively regulated by estrogen through the AP1 pathway, the significant increase in laying hens compared with that in pre-laying birds and higher levels in CEP birds than in IEP birds in both liver and ovary might be regulated by some other unknown factors besides estrogen. The expression patterns of *ACSF2* matched the egg-laying curve well. The findings in this study pave the way for further exploration of the functions of *ACSFs* in the regulation of liver lipid metabolism and egg-laying performance in chicken. Additional investigation on the biological pathways of *ACSFs* should deepen our understanding of the lipid metabolism mechanism in chicken liver and promote reproductive performance in the chicken industry.

## Materials and Methods

### Ethics statement

To ensure animal welfare, the experiment was strictly performed in accordance with protocols approved by the Institutional Animal Care and Use Committee (IACUC) of Henan Agricultural University. All experimental animals were female Lushi green-shell-egg chickens from the Animal Center of Henan Agricultural University and were maintained on *ad libitum* food and water under the same conditions.

### Experimental animals and samples preparation

To investigate temporal and spatial expression patterns of *ACSFs*, eight healthy female birds were humanely slaughtered at hatching and at ages of 1, 10, 20, 30, 35 and 50 weeks. Tissues including heart, liver, spleen, lung, kidney, pectoralis, glandular stomach, pancreas, abdominal fat, duodenum and ovary were quickly removed, snap-frozen in liquid nitrogen and stored at −80 °C until use.

To investigate the correlation between the expression level of *ACSFs* and egg-laying performance, 16 healthy female chickens at the age of 50 weeks were selected from the production stock of the layers. Among them, eight hens were continuously producing eggs and the other eight had stopped for the last 2 weeks. The former were designated as the continuous egg production hen (CEP) group and the latter as the inactive egg production hen (IEP) group. The birds were humanely slaughtered, then liver and ovary samples were collected as described above. In our study, the ovary with pre-hierarchal small white follicle was used.

To study the effects of estrogen on the expression of *ACSF* subtypes, a total of 40 female chickens at an age of 10 weeks were divided randomly into four groups (10 birds per group). The birds in the first three groups were intramuscularly injected with 17β-estradiol at a dose of 0.5, 1.0 or 2.0 mg/kg (Sigma, St. Louis, MO, USA) dissolved in olive oil. The other 10 birds were intramuscularly injected with the same amount of olive oil as a control group. After 12 h, all of the chickens were killed and their livers were processed as mentioned above.

### Chicken embryonic primary hepatocytes culture and treatment

The isolation and culture of chicken embryonic primary hepatocytes were carried out using our method^42^ adapted from Fischer and Marks^43^. In brief, a total of 25 chicken embryos were obtained by the incubation of specific pathogen-free eggs for 18 days under standard conditions. The embryonic livers were removed, minced and washed in 1 × phosphate-buffered saline (PBS) (Solarbio, Beijing, China), and then digested by collagenase IV for 7–15 min at 35 °C. The cells were filtrated with a 100-, 200- and 500-mesh sieve in turn, collected by centrifugation at 98 rcf for 5 min at room temperature and washed with 1 × PBS three times after each filtration. The washed cells were then resuspended by effective Percoll comprising Percoll stoste (Solarbio, Beijing, China), 10 × PBS and 1 × PBS at a ratio of 2.7:0.3:2, and collected by centrifugation at 885 rcf for 15 min at room temperature. Then, DMEM/F-12 media replenished cells with 10% foetal bovine serum (FBS) (Gibco, Australia) and 2% penicillin-streptomycin (Gibco, Australia). The cell density was adjusted to 5 × 10^5^ cells/mL using a Luna automated cell counter (Biosystems L10001, South Korea) and cells were cultured in six-well plates at 37 °C and 5% CO_2_. The cells were serum-starved for 6 h when they had grown to 80–90% confluence and then randomly divided into seven groups (six repeats for each group). The cells in groups 1, 2 and 3 were treated with 25, 50 and 100 nM 17β-estradiol dissolved in ethyl alcohol, respectively. The cells in groups 4, 5 and 6 were first treated with 1 µM ER subtype antagonists, 1,3-bis(4-hydroxyphenyl)-4-methyl-5-[4-(2-piperidinylethoxy)phenol]-1H-pyrazoledihydrochloride (MPP), tamoxifen (TAM) and ICI 182,780 (ICI) (Sigma, St. Louis, MO, USA), respectively, for 4–6 h, and then 17β-estradiol was added to a final concentration of 100 nM, followed by incubation for 12 h. The cells were washed in 1 × PBS, collected with TRIzol® reagents (Takara, Kyoto, Japan) and stored at −80 °C until use. The cells in group 7, which served as a control, were treated with the same amount of solvent only.

### RNA extraction and reverse transcription

Total RNA was extracted from tissues and cells using TRIzol® reagents (Takara, Kyoto, Japan). The purity and concentration were measured using NanoDrop2000 (Thermo Scientific, Wilmington, DE, USA) and RNA integrity was determined by denaturing agarose gel electrophoresis. The RNA samples with OD260/280 ratios above 1.8 and sharp, bright 28 S and 18 S bands in denaturing agarose gel were selected for further analysis. The RNA was reverse-transcribed into cDNA with random hexamers by using PrimerScript^TM^ RT Reagent Kit (Takara, Kyoto, Japan), in accordance with the manufacturer’s instructions. The cDNA was stored at −20 °C until use.

### Quantitative real-time PCR (qPCR)

To examine the relative expression levels of genes, a SYBR green-based qPCR analysis was conducted on a LightCycler®96 Real-Time PCR system (Roche Applied Science). The primers were designed via Primer 5.0 and synthesized by Sangon Biotech (Shanghai, China) (Table S1). All of the primers used in our study were designed according to the same coding sequences among all alternative splice variants and had to span an exon–exon junction. The qPCR was carried out in a 10-μL reaction volume consisting of 5 μL of 2 × SYBR®Premix Ex Taq™ II (Takara, Kyoto, Japan), 0.5 μL each of forward and reverse primers (10 μM), 1 μL of cDNA and 3 μL of RNase-free water. The housekeeping gene *β-actin* served as an internal control for normalization. All reactions were performed in triplicate. The qPCR conditions were as follows: an initial 5 min of pre-denaturing at 95 °C; followed by 40 cycles of 30 s of denaturing at 95 °C, 30 s of annealing at 60 °C and 30 s of extension at 72 °C; then a further 10 min of extension at 72 °C. The specificity of each primer pair was validated by melting curve analysis. The relative expression of genes was calculated by the 2^−∆∆Ct^ method.

### Sequence bioinformatic analysis

Nucleotide and amino acid sequences were identified using Ensembl (http://www.ensembl.org/index.html) and NCBI (https://www.ncbi.nlm.nih.gov/). PhyloViewin Genomicus v71.01 (http://www.genomicus.biologie.ens.fr/genomicus-71.01/cgi-bin/search.pl) was used for consensus conserved genomic synteny. Amino acid sequence alignment was performed by DNAMAN. The phylogenetic tree was constructed using the neighbour-joining method in Molecular Evolutionary Genetics Analysis version 6.0 (MEGA6.0) based on amino acid sequence alignments generated from ClustalW. SMART (http://smart.embl-heidelberg.de/) was employed for protein functional domain prediction. The program Find Individual Motif Occurrences (FIMO) (http://meme-suite.org/tools/fimo) was used to predict the functional motifs in amino acid sequences and the estrogen binding sites in the promoter region of genes.

### Statistical analysis

The data are expressed as mean ± standard deviation of the mean. Statistical significance between three or more experimental groups was evaluated by one-way ANOVA followed by Dunnett’s test for multiple comparisons by SPSS23.0 (IBM, Chicago, IL, USA). Statistical significance between two experimental groups was evaluated by T-test for comparisons by SPSS23.0 (IBM, Chicago, IL, USA). The graphics were drawn using GraphPad Prism 5 (GraphPad, San Diego, CA, USA). P-value < 0.05 was considered statistically significant, and P-value < 0.01 was considered extremely significant.

## Electronic supplementary material

supplementary information
