# Supplementary material for: Dynamic Expression Profile, Regulatory Mechanism and Correlation with Egg-laying Performance of ACSF Gene Family in Chicken (Gallus gallus)
Source: Sci Rep. 2018 May 31;8:8457. doi: 10.1038/s41598-018-26903-6 (PMC5981300; doi:10.1038/s41598-018-26903-6)
Supplement: Supplementary file 1 — supplementary information [file 41598_2018_26903_MOESM1_ESM.pdf]

Dynamic Expression Profile, Regulatory Mechanism and Correlation with Egg-laying Performance of *ACSF* Gene Family in Chicken (*Gallus gallus*)

Weihua Tian[1, ¶], Hang Zheng[1, ¶], Liyu Yang[1], Hong Li[1,2,3], Yadong Tian[1,2,3], Yanbin Wang[1,2,3], Shijie Lyu[4], Gudrun A. Brockmann[4], Xiangtao Kang[1,2,3]*, Xiaojun Liu[1,2,3]*

[1]College of Animal Science and Veterinary Medicine, Henan Agricultural University, Zhengzhou 450002, China

[2]Henan Innovative Engineering Research Center of Poultry Germplasm Resource, Zhengzhou 450002, China

[3]International Joint Research Laboratory for Poultry Breeding of Henan, Zhengzhou 450002, China

[4]Albrecht Daniel Thaer-Institute of Agricultural and Horticultural Sciences, Humboldt-Universit€at zu Berlin, Invalidenstraße 42, Berlin 10115, Germany.

¶These authors contributed equally to this work.

*Corresponding authors.

Xiaojun Liu: xjliu2008@hotmail.com

Xiangtao Kang: xtkang2001@263.net

Supplementary information

a

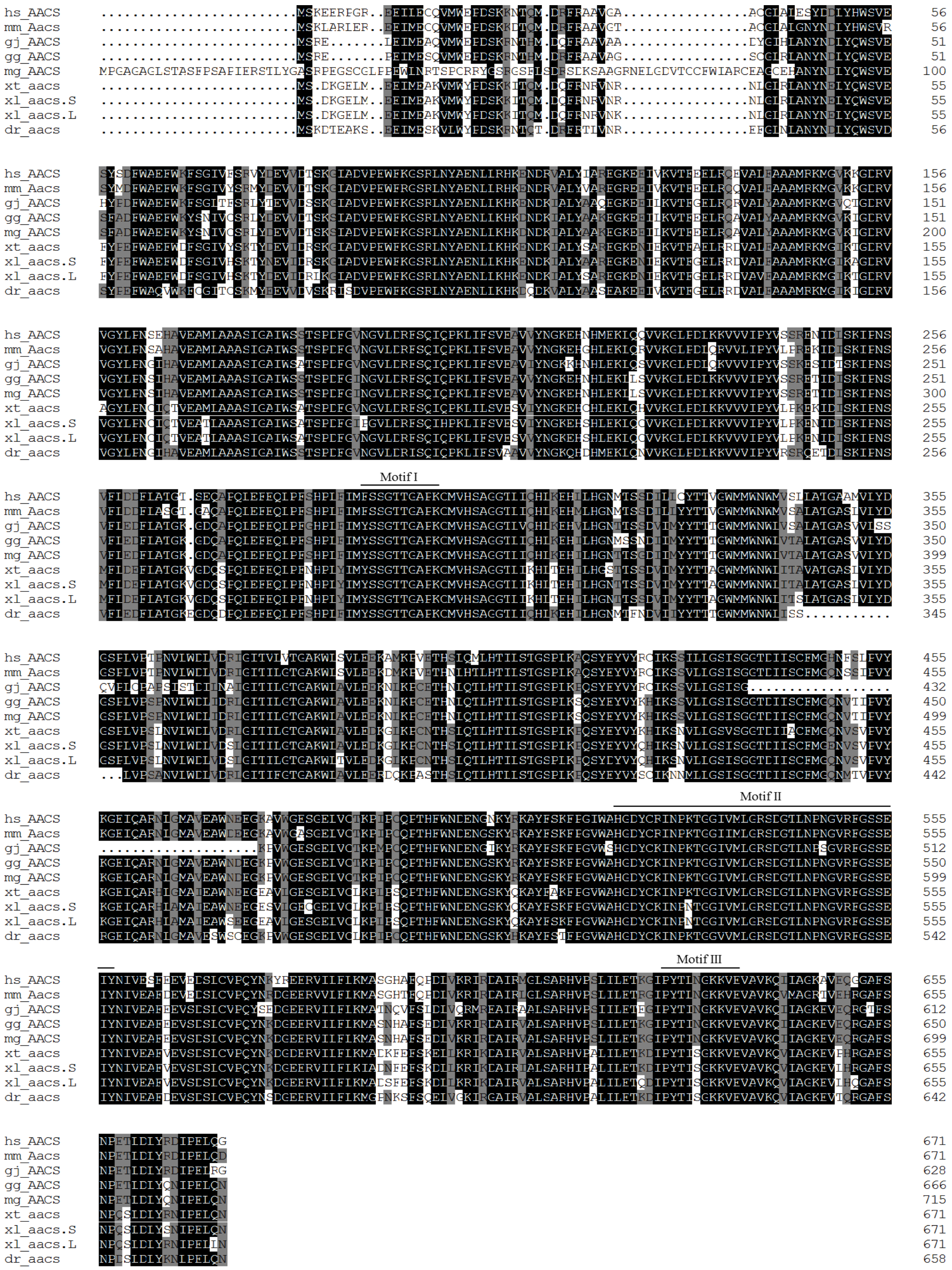

b

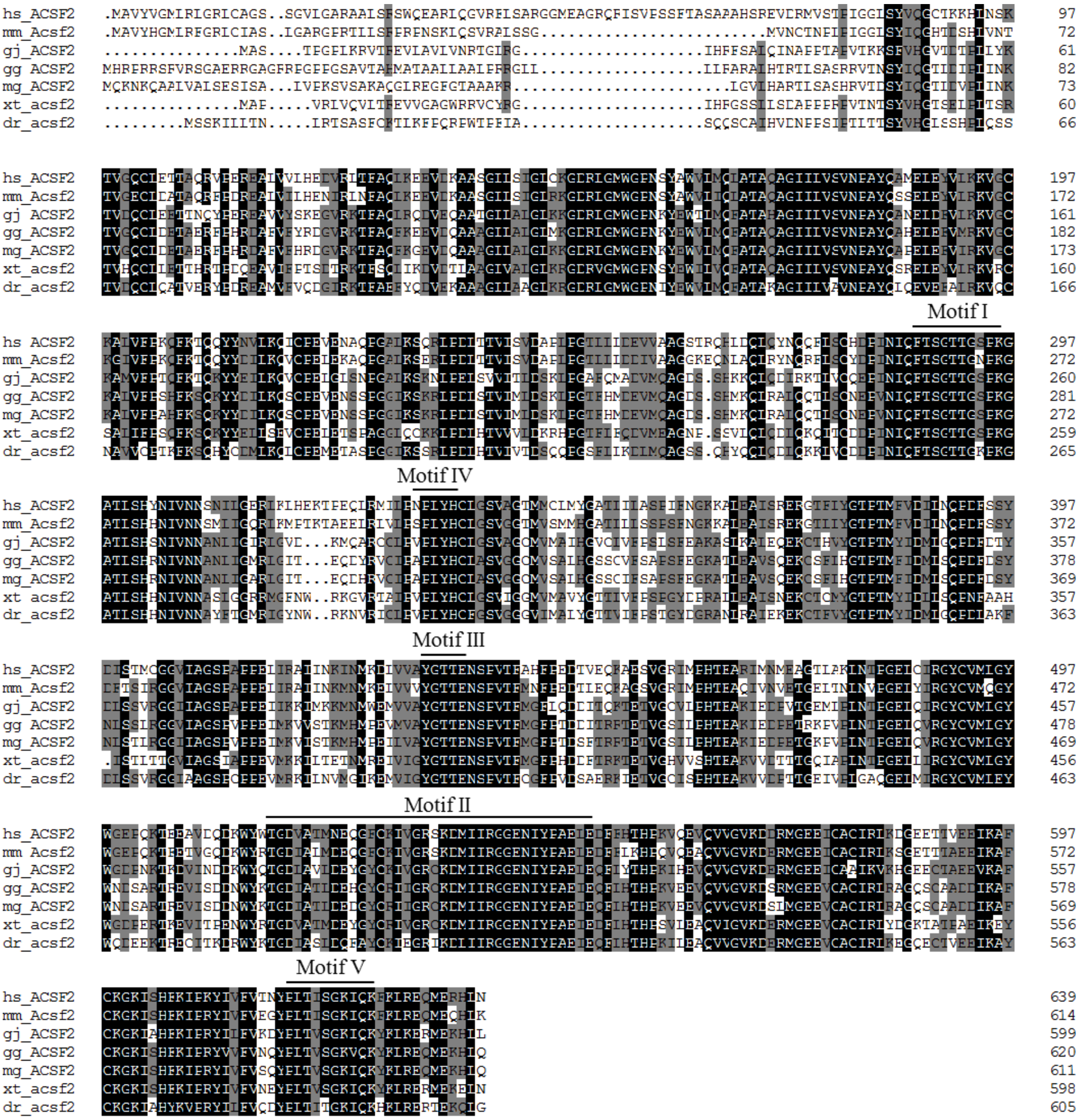
hs_ACSF2
mm_Acsf2
gj_ACSF2
gg_ACSF2
mg_ACSF2
xt_acsf2
dr_acsf2
Motif I
Motif IV
Motif III
Motif II
Motif V

c

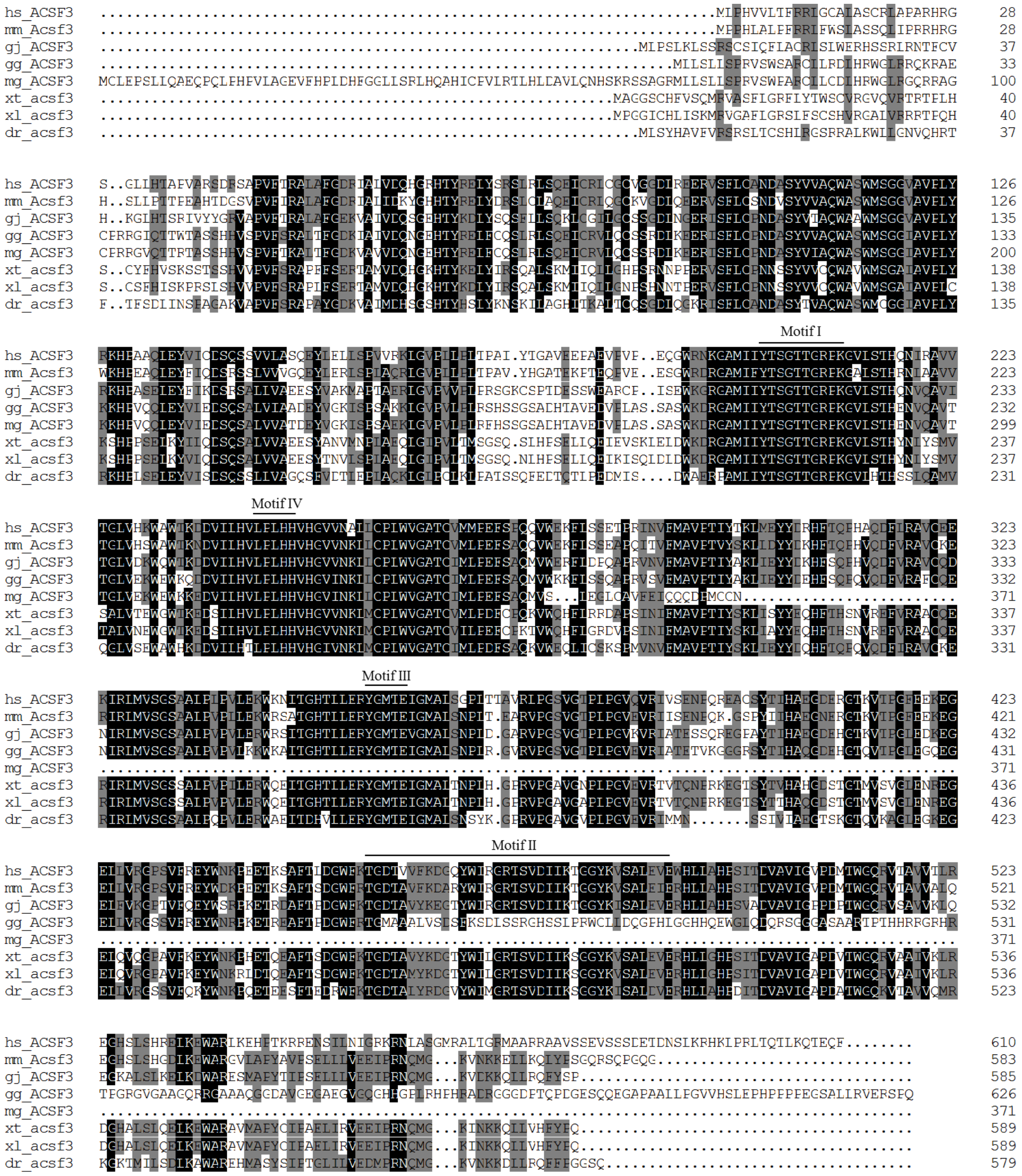
hs_ACSF3
mm_Acsf3
gj_ACSF3
gg_ACSF3
mg_ACSF3
xt_acsf3
xl_acsf3
dr_acsf3
Motif I
Motif IV
Motif III
Motif II

d

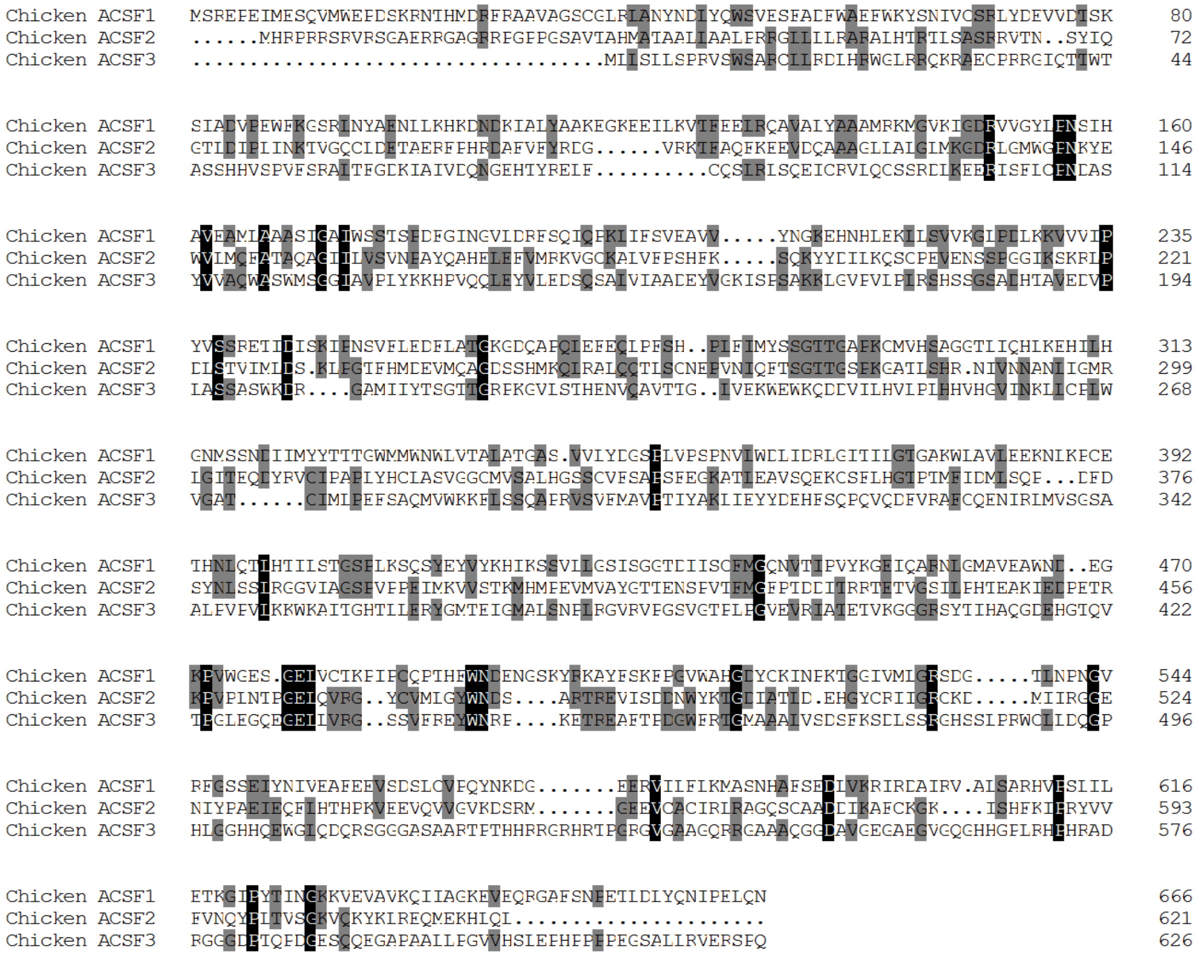

Figure S1. Multiple alignments of ACSF1, ACSF2 and ACSF3 amino acid sequences among different species by DNAMAN. 50%–75% identity sites are shaded by gray and more than 75% identity sites are shaded by black. (a) Multiple alignments of ACSF1 amino acid sequences among different species. The amino acid sequences name and NCBI accession numbers used in the multiple alignment were as follows: hs AACS, Homo sapiens (human), NP_076417.2; mm Aacs, Mus musculus (mouse), NP_084486.1; gj AACS, Gekko japonicas (gecko) , XP_015276338.1; gg AACS, Gallus gallus (chicken), NP_001006184.1; mg AACS, Meleagris gallopavo (turkey), XP_010718535.1; xl aacs.S, Xenopus laevis (African clawed frog), XP_018099672.1; xl aacs.L, Xenopus laevis (african clawed frog), XP_018117990.1; xt aacs, Xenopus tropicalis (western clawed frog), NP_001039244.1; dr aacs, Danio rerio (zebrafish), NP_957303.1; (b) Multiple alignments of ACSF2 amino acid sequences among different species. The amino acid sequences were named as above, the NCBI accession numbers were as follows: hs ACSF2, NP_001275897.1; mm Acsf2, NP_722502.1; gj ACSF2, XP_015266223.1; gg ACSF2, XP_015151000.1; mg ACSF2, XP_010719920.1; xt acsf2, XP_002939621.2; dr acsf2, NP_001132910.1; (c) Multiple alignments of ACSF3 amino acid sequences among different species. The NCBI accession numbers were as follows: hs ACSF3, XP_011521244.1; mm Acsf3, NP_659181.2; gj ACSF3, XP_015262215.1; gg ACSF3, XP_425134.4; mg ACSF3, XP_010716483.2; xl acsf3, NP_001086314.2; xt acsf3, NP_001121382.2; dr acsf3, XP_021329817.1; (d) Amino acid sequences alignment of chicken ACSF1, ACSF2 and ACSF3.

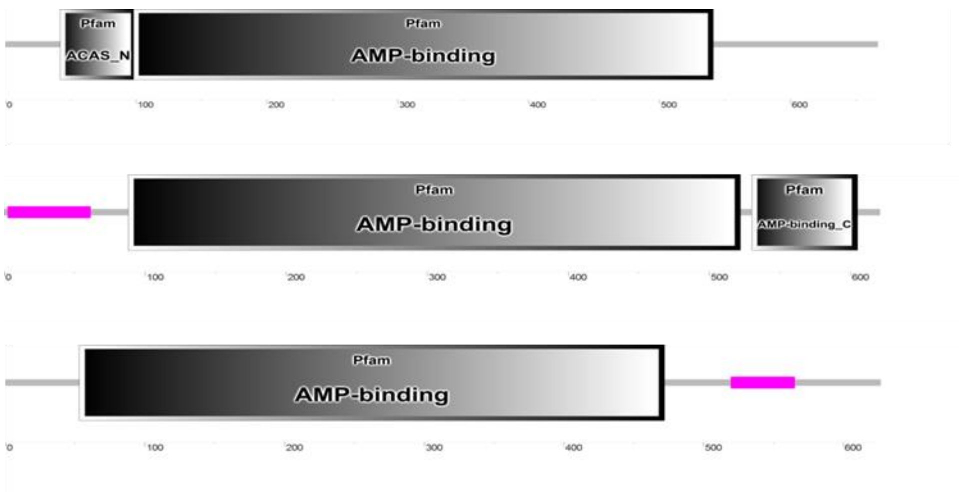

Figure S2. Functional domains analysis of chicken ACSF1, ACSF2 and ACSF3 amino acid sequences

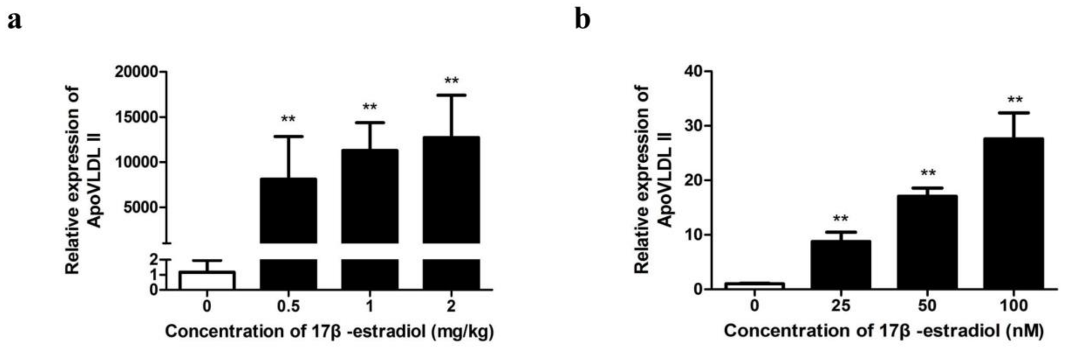

Figure S3. Effects of different doses of 17 β-estradiol on the mRNA expression of *ApoVLDL II* in chicken liver tissues and primary hepatocytes. (a) Effects of different doses of 17 β-estradiol on the mRNA expression of *ApoVLDL II* in chicken liver tissues. (b) Effects of different doses of 17 β-estradiol on the mRNA expression of *ApoVLDL II* in chicken primary hepatocytes. The housekeeping gene *β-actin* was used as an internal standard to estimate mRNA relative expression. Data were represented as Mean ± SD (n = 8). *significant differences ($P < 0.05$), **highly significant differences ($P < 0.01$).

Table S1 The primer sequences and product lengths for qPCR

gene	Primer sequence	Product (bp)
ACSF1	F: TTAAAGGGAGCCGCCTGAAC; R: GCAGCATACAAAGCTACGGC	148
ACSF2	F: TTATAGGCCGCTGCAAGGAC; R: ATCCGCGAATCCTTCACTCC	130
ACSF3	F: CCTACACCATCCACGCTCAG; R: TGAACCATCCATCGGGTGTG	162
ApoVLDL II	F: CAATGAAACGGCTAGACTCA; R: AACACCGACTTTTCTTCCAA	108
β-actin	F: CAGCCAGCCATGGATGATGA; R: ACCAACCATCACACCCTGAT	147

Note: F refers to forward primer, R refers to means reverse primer.

Table S2 The detailed genomic locations of *ACSF1*/*AACS* genes and neighbouring genes among different species

Species	Symbol	Chromosome location	Strand	Start	Stop
Human	SCARB1	12	-	124777628	124863973
	UBC	12	-	124911645	124915041
	DHX37	12	-	124946824	124989125
	BRI3BP	12	+	124993648	125051845
	AACS	12	+	125065367	125143333
	TMEM132B	12	+	125186750	125662377
	TMEM132C	12	+	128267170	128707915
	SLC15A4	12	-	128793194	128823996
	GLT1D1	12	+	128853323	128984964
Mouse	Scarb1	5	-	125277087	125341094
	Ubc	5	-	125385965	125390017
	Dhx37	5	-	125413747	125434145
	Bri3bp	5	+	125441538	125460885
	Aacs	5	+	125475873	125517403
	Tmem132b	5	+	125532418	125792583
	Tmem132c	5	+	127241826	127565790
	Slc15a4	5	-	127595478	127632900
	Glt1d1	5	+	127632130	127708750
Chicken	SCARB1	15	+	4508303	4524546
	DHX37	15	+	4489954	4501936
	BRI3BP	15	+	4485386	4486760
	AACS	15	-	4444355	4481664
	TMEM132B	15	-	4261071	4426221
	TMEM132C	15	-	3795221	3975758
	SLC15A4	15	+	3750314	3771648
	GLT1D1	15	-	3704710	3746830
Turkey	SCARB1	17	+	4582554	4595819
	DHX37	17	+	4559934	4574334
	BRI3BP	17	+	4557658	4562058
	AACS	17	-	4516557	4556825
	TMEM132C	17	-	3876699	4012333
	SLC15A4	17	+	3828842	3850357
	GLT1D1	17	-	3783038	3825524
Frog	scarb1	GL172777.1	-	533460	561717
	dhx37	GL172777.1	-	621133	639770
	bri3bp	GL172777.1	+	646971	657947
	aacs	GL172777.1	+	671479	712055
	TMEM132B	GL172777.1	+	1051224	1063166
	tmem132c	GL172777.1	+	1512252	1697149
	slc15a4	GL172777.1	-	1776696	1789521

	glt1d1	GL172777.1	+	1799975	1868989
Lizard	SCARB1	LGb	+	1342765	1373321
	DHX37	LGb	+	1313349	1328807
	BRI3BP	LGb	+	1298637	1308580
	AACS	LGb	-	1270638	1298603
	TMEM132B	LGb	-	983503	1243996
	TMEM132C	LGb	-	570551	726430
	SLC15A4	LGb	+	526496	556913
	GLT1D1	LGb	-	485310	522604
Coelacanth	SCARB1	JH126594.1	+	789399	819981
	DHX37	JH126594.1	+	737367	765587
	BRI3BP	JH126594.1	-	663101	671888
	AACS	JH126594.1	-	590031	650329
	TMEM132B	JH126594.1	-	474378	475529

142
